# Ammonia as a Hydrogen Carrier: Energetic Assessment of Processes Integrated with Fuel Cells for Power Generation

**DOI:** 10.1021/acs.energyfuels.4c04626

**Published:** 2025-01-24

**Authors:** Maria Portarapillo, Augusto Bellucci Sessa, Danilo Russo, Almerinda di Benedetto

**Affiliations:** Department of Chemical Engineering, Materials, and Industrial Production, University of Naples Federico II, P. le V. Tecchio 80, 80125 Napoli, Italy

## Abstract

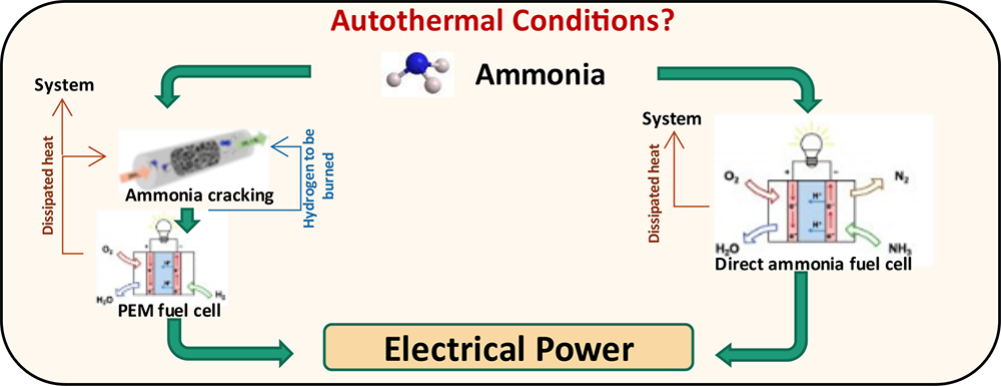

In the context of the near-future hydrogen economy, ammonia is regarded as one of the most promising hydrogen carriers in the short-to-medium term. As part of the broader transition to a new energy paradigm, the well-established and extensive ammonia infrastructure can serve as a platform for green hydrogen transportation, storage, and utilization. This study analyzes various process configurations integrated with different types of fuel cells for ammonia utilization through Aspen Plus simulations. The evaluation focuses on overall energetic efficiency (ranging from 31.20 to 51.50% depending on the adopted configuration), autothermality (42.00 to 100.00%, based on the adopted process configuration), and emissions from external heat sources (0.03–0.07 kg_CO2_/kWh). Assessments are conducted parametrically across different fuel cell efficiencies (50.0–65.0%). Results suggest that high-temperature PEMFC and direct ammonia solid oxide fuel cells (SOFCs) offer a balance between overall efficiency (40.2–51.5 and 35.00–52.0%, respectively) and feasibility of achieving autothermal operations under nitrogen dilution (up to 25.0%). Considering technological maturity and operational lifespan, high-temperature PEMFCs and SOFCs emerge as a promising component for such integrated systems.

## 1 Introduction

Hydrogen is expected to play a pivotal role in the near future as part of the green transition.^1−3^ Governments and industries are focusing on hydrogen as a key energy vector and fuel for decarbonization due to its high energy density per mass (120 MJ/kg), higher than fossil fuels, its potential for production from renewable sources, and its oxidation resulting in net-zero emissions.^1,4^ Hydrogen is a clean energy carrier that does not emit climate-altering gases such as CO_2_, or substances harmful to human health like benzene or particulate matter.^5^ These attributes make it a viable solution for energy storage^6^ and a sustainable option for generating clean electricity when utilized in fuel cells (FCs).^7^ As a result, the development of hydrogen technologies is driven by several advantages. However, the decarbonization potential of hydrogen critically depends on the technologies used for its green production from renewable sources.^8^ Additionally, it is important to address hydrogen inherent risks, as it is classified as an extremely flammable gas. Accidental releases, such as those caused by tank rupture or breaches, can lead to explosions and fires, posing safety concerns.^9−11^ With hydrogen increasingly recognized as a cornerstone of decarbonization efforts, FCs are emerging as vital components for future mobile and stationary applications.^12^ FCs enable the generation of electricity without emitting pollutants. However, hydrogen-powered FCs require hydrogen storage, which often involves extreme conditions, such as cryogenic temperatures or high pressures. These storage requirements can be costly and may pose safety challenges in the event of an accident.^13,14^

To address challenges associated with hydrogen use, researchers have investigated alternative carriers, such as methanol,^15^ ethanol,^16^ glycerol,^17^ and ammonia,^18,19^ that can release hydrogen while reducing transportation and storage issues, leveraging existing large-scale infrastructures.

Among these options, carbon-based fuels are less desirable because they emit carbon dioxide during both synthesis and energy conversion. In contrast, ammonia, a carbon-free molecule, represents a viable alternative. Its lower heating value is 8.9 kWh/kg, higher than methanol (6.2 kWh/kg) but lower than diesel (13.2 kWh/kg).^20^ During ammonia oxidation, nitrogen is released back into the atmosphere, completing the carbon-free cycle.^21,22^ Research is ongoing into green ammonia synthesis methods, such as electrochemical synthesis, which operates under lower temperature and pressure conditions.^23^ Ammonia can be stored in liquid form at room temperature under a pressure of 8.6 bar.^24^ One mole of ammonia contains 1.5 mol of hydrogen, corresponding to 17.8% hydrogen by weight. In terms of volumetric density, ammonia stores 108 kg H_2_/m^3^, four times the density achieved with alternative hydrogen storage carriers, such as metal hydrides (25 kg H_2_/m).^3,25^ This technique mitigates significant infrastructural challenges associated with H_2_ distribution and reduces safety risks and explosion hazards of direct storage of hydrogen at pressures ranging from 350 to 700 bar.^24^ In terms of flammability, ammonia is safer, with a much narrower flammability range (17–27% in air) compared to hydrogen (4–75% in air).^20^ However, ammonia poses a toxicity risk, as concentrations exceeding 2500 ppm can be fatal after 30 min of exposure.^20,26^ Therefore, ammonia is a cost-effective fuel compared to hydrogen for storage and transportation^27^ thanks to its well-established global production and distribution infrastructure, ensuring a continuous fuel supply. Based on the above considerations, NH_3_ seems to be one of the most promising energy hydrogen carriers, at least in the short-to-medium term. In the context of electrical power generation via FCs, ammonia can be directly fed into solid oxide FCs (SOFC) or decomposed into hydrogen and nitrogen for use in hydrogen FCs. Ammonia reforming offers two primary advantages over hydrocarbons reforming. First, the gas purification step is simpler since ammonia decomposes into hydrogen and nitrogen. Second, unlike hydrocarbons and methanol reforming, the decomposition occurs in a single step with a single feed stream, resulting in significant cost savings.^28^

NH_3_ decomposition is an attractive fuel processing technology in FC applications.^29^ The endothermic catalytic cracking of ammonia is one of the most energy-efficient commercial processes.^20^ Common catalysts include iron oxide, molybdenum, ruthenium, and nickel.^30−32^ Unlike synthesis, cracking does not require high pressures and typically operates at temperatures around 600–700 °C.^31^ The decomposition reaction is as follows:
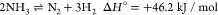


The use of ammonia for electric power generation can be achieved by integrating ammonia-systems with various types of FCs, such as high-temperature proton exchange membrane FC (HT-PEMFC), low-temperature PEMFC (LT-PEMFC), and direct-ammonia or hydrogen solid oxide FC (SOFC). PEMFCs are of significant interest due to their high power density (39.7 kW/kg), efficiency (40–60%), and rapid startup at low operating temperatures.^33^ HT-PEMFCs offer several advantages over LT-PEMFCs. These include improved tolerance to impurities, easier heat rejection, enhanced electrode kinetics, simplified water management, and possible cost reduction from adoption of non-noble catalysts. However, they also present challenges such as increased degradation rates and longer startup time. Specifically, the degradation of membranes and catalysts, as well as carbon corrosion, are key issue the reduce lifespan of HT-PEMFCs (typically ranging from 8000 to 20,000 h),^34^ particularly at higher operating temperatures and under dynamic conditions.^35^ Despite these challenges, HT-PEMFCs demonstrate a performance advantage over LT-PEMFCs when operating with mixtures rather than pure H_2._^36,37^ Specifically, HT-PEMFCs demonstrate greater tolerance to nitrogen dilution at the anode without a significant loss in energy efficiency. Modeling studies conducted on NH_3_,^36^ supported by experimental evidence, indicate a correlation between the potential difference across HT-PEMFCs electrodes and the H_2_/N_2_ ratio within the FC. In general, nitrogen in the feed stream negatively impacts the polarization curve of the FC. However, at nitrogen dilution levels ranging from 0 to 20% and temperature between 140 and 160 °C, FC performance remains relatively constant. At high nitrogen dilution levels (30–50%), temperature exert a significant influence on FC performance.^36^ These findings suggest that increased nitrogen dilution amplifies the sensitivity of FC performance to temperature variations. Therefore, managing both temperature and dilution levels simultaneously is critical for optimizing the operation of HT-PEMFC systems (Table 1). Temperature generally enhances performance across most nitrogen dilution concentrations, aligning with observations from polarization curves. However, as nitrogen concentration increases, cell impedance also rises, becoming particularly pronounced at a nitrogen concentration of 50%.

**Table 1:** Summary Table of Polarization Curves,^36^ Current Density 0.25 A/cm^2^

temperature (°C)	*E* (V) H_2_/N_2_ = 100/0	*E* (V) H_2_/N_2_ = 80/20	*E* (V) H_2_/N_2_ = 60/40	*E* (V) H_2_/N_2_ = 50/50
180	0.629	0.621	0.616	0.606
160	0.625	0.612	0.607	0.593
140	0.615	0.607	0.590	0.573

Previous studies^37^ have shown that in LT-PEMFCs, a significant decrease in electrode potential (approximately 20% at a current density of 0.25 A/cm^3^) occurs when transitioning from pure H_2_ to a mixture of 80% H_2_ and 20% N_2_ (Table 2).

**Table 2:** Summary Table Polarization Curves Comparison between HT-PEMFC and LT-PEMFC,^36,37^ Current Density 0.25 A/cm^2^

temperature (°C)	*E* (V) H_2_/N_2_ = 100/0	*E* (V) H_2_/N_2_ = 80/20
160	0.625	0.612
60	0.554	0.440

Additionally, it has been observed that PEMFCs suffer substantial performance losses when trace amounts of NH_3_ react with H^+^ ions within the membrane and catalytic layers.^38^

To remove small concentrations of gaseous NH_3_, it can react by passing through packed liquid or solid bed reactors.^39^ Solid alkali chloride salts, such as MgCl_2_, can react with NH_3_ to form metal-NH_3_-chloride compounds, absorbing 1.07 g of NH_3_ per gram.^39^ Phosphoric acid (H_3_PO_4_) is another option, absorbing up to 0.53 g of NH_3_ per gram. Silica materials in various forms, such as beads or gels, can adsorb NH_3_ depending on factors surface acidity, specific surface area, and water content.^39^ The selection of the ad/ab-sorption method depends on the specific application. Hunter et al.^40^ studied H_2_ production for powering a small PEMFC using MgCl_2_. Zhang et al. demonstrated that a feasible amount of commercially available adsorbent (such as Mesosystems Technology) can reduce ammonia concentration to trace levels lower than 1 ppb.^20^ However, it should be noted that these adsorption methods do not remove N_2_.^39^

SOFCs directly fed with ammonia theoretically offer the highest efficiencies (55–65%) and eliminate the need for an external ammonia cracking reactor, leveraging the autothermal nature of the process.^41^ However, direct ammonia FCs face critical challenges in responding to load changes.^42^ Dwivedi^43^ conducted a review focusing on SOFC technology, highlighting the high operational temperatures required (typically 500–1000 °C). These temperatures necessitate specialized materials with high-temperature tolerance and complex thermal management systems. Consequently, system complexity and associated costs present significant challenges. Additionally, prolonged operation results in degradation of critical components, such as electrodes and electrolytes, causing gradual performance declines. As a result, reported degradation rates range from 1.0 to 4.0% per 1000 h of operation, which is 5–20 times higher than the target set by the U.S. Department of Energy.^44,45^ Moreover, power generation efficiency is intricately influenced by the specific reformate used for hydrogen production, which directly affects the feed composition and hydrogen concentration.^46^ This variability, among other factors, explains the broad efficiency ranges reported for these devices. Despite these limitations, ongoing research aims to mitigate these challenges and enhance the efficiency and commercial viability of SOFCs.

Currently, no systematic comparison exists in the literature for ammonia-based integrated systems with heat recovery and various types of FCs. Most state-of-the-art energy studies focus on the direct use of ammonia in SOFC^17,47−49^ and strategies to enhance the overall efficiency of integrated systems by utilizing residual heat in gas turbines and/or thermodynamic cycles such as Kalina Cycles. These studies report optimized efficiencies ranging from 60 to 70%. A recent pioneering study^50^ investigated the efficiency of integrated systems with HT-PEMFCs, reporting an overall efficiency of 40.1% under specific conditions: a feed composition of 60% H_2_ and 40% N_2_ to the anode; a utilization factor (*U*_f_) of 52%, and no heat recovery from the cathodic exhaust stream. However, the strategies employed in these studies focused on optimizing single configurations and were carried out under varying conditions, which do not allow for a fair comparison between different process configurations. Building on these considerations, this work aims to evaluate the energy efficiency of different integrated configurations by conducting parametric studies of the most influential operating conditions in their common operating ranges, considering the wide range of factors affecting fuel cell performances. The analyzed configurations include: (i) coupling a catalytic NH_3_ decomposition system with LT-PEMFC, HT-PEMFC, or hydrogen SOFC; (ii) systems utilizing direct-ammonia SOFC. As a result, this work aims to facilitate a fair comparison and establish a benchmark for various systems across a range of conditions through parametric analysis. Additionally, this study lays the groundwork for further integration of components, enabling differentiation between the contributions of coupling cracking units with FCs of varying performances and the contributions of additional components, such as gas turbines and thermodynamic cycles.

In all configurations, heat recovery is also incorporated. An indexing system is defined to evaluate energy efficiency.

## 2 Methods

### 2.1 Conceptualization and Assumptions

**Figure 1:**
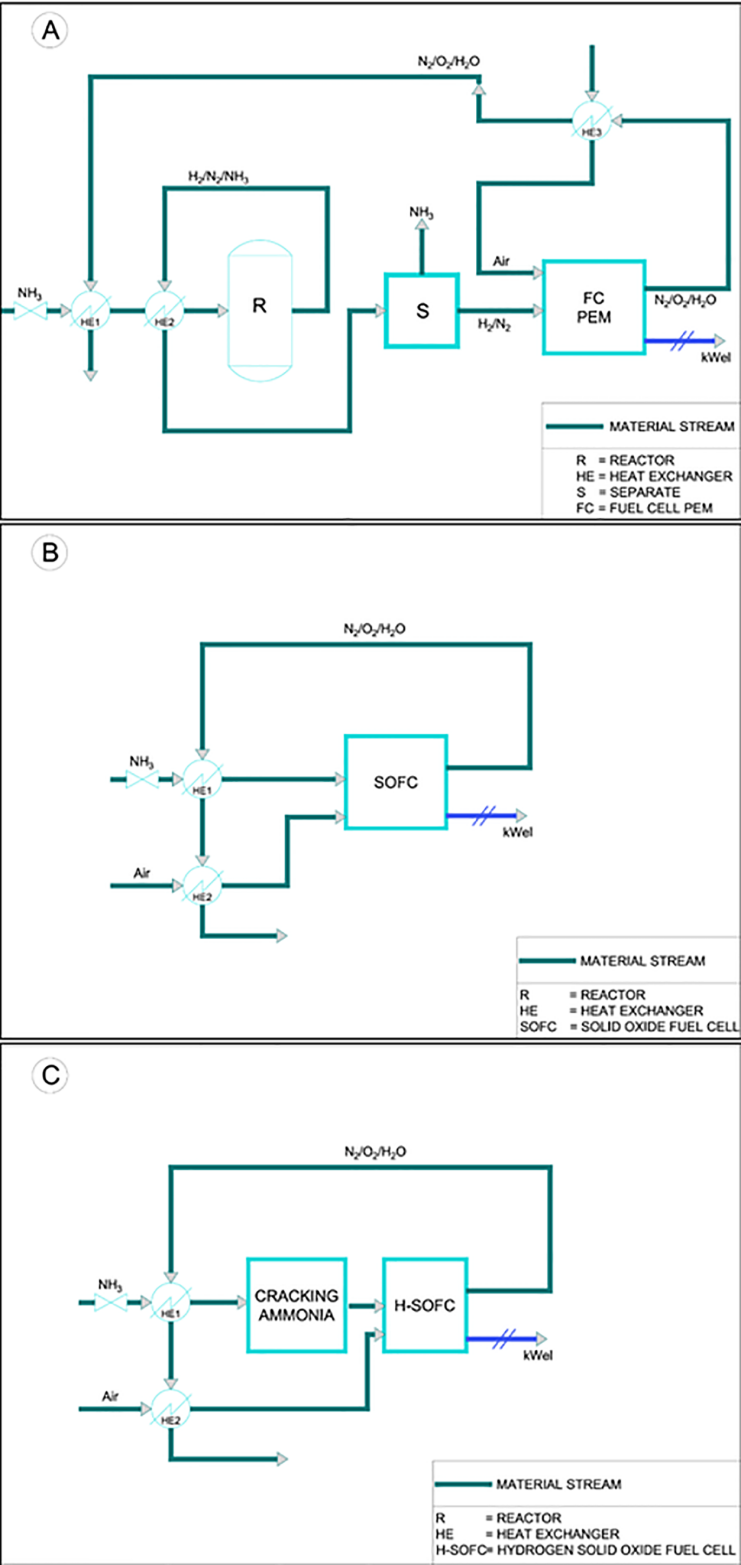
Process diagram for PEMFC (A), direct-ammonia SOFC (B), and hydrogen SOFC (C) systems.

A general scheme of adopted configurations in provided in Figure 1. Process was conceptualized as follows. Ammonia is transferred from a storage tank through a pipeline and passed through a pressure-reducing valve, lowering its pressure from approximately 9 bar to the reactor working pressure (1 bar). Subsequently, the ammonia stream is preheated in a heat exchanger. The temperature of the stream exiting the heating block is determined by energy balancing to ensure self-sustained operation, where the system thermal energy offsets its heat requirements. The system comprises four heat streams: (i) the heat required for the preheating block upstream of the reactor, (ii) the heat released from the FC, including the heat that can be recovered from unconverted hydrogen in the anode outlet (iii) the heat required for the ammonia decomposition reaction, (iv) and the heat released from a cooling block downstream of the reactor, used to adjust the outlet stream temperature to the FC operating conditions. To evaluate the extent of partial or total heat recovery, these heat fluxes were analyzed combined. The heat needed to sustain the reactor was either recovered from other sections of the plant or supplied by external methane combustion, chosen as a benchmark to evaluate associated CO_2_ emissions, depending on the specific configuration considered.

Aspen Plus^51^ was employed to perform energetic assessments. This widely adopted software is commonly used to simulate process operations, evaluate performance, and optimize processes using built-in mathematical and thermodynamic models.^51^ The primary objective of the energy evaluations was to identify conditions for autothermal operation or partial heat recovery.^52^ In the simulations, the ideal gas equation was employed as the standard equation of state model to evaluate the properties of the species. This approach is justified by the system operating conditions, which satisfy the assumptions of ideality. In this study, an equilibrium reactor was used to simulate ammonia decomposition. The ammonia-cracking reactor is modeled as an R-GIBBS reactor, where the product composition is determined by minimizing the Gibbs free energy, subject to atomic balance constraints. This approach aligns with real-world applications; as highlighted in technological studies, industrial-scale ammonia cracking is typically conducted at near-complete conversion under elevated temperatures and moderate pressures.^54^ Available reports from industry also demonstrate that catalysts are designed to operate near their full potential, achieving conversion levels that align closely with thermodynamic limits at high temperatures and low pressures.^55^ Comprehensive reviews on catalysts indicate a wide range of conversions achieved with various catalytic systems, including some that reach full conversion.^53^ Innovative catalytic systems are pushing these limits further, with reports of complete conversion at temperatures as low as 450 °C.^56^ However, it is crucial to emphasize that most lab-scale catalytic studies focus on metrics such as turnover frequency (TOF), turnover number (TON), and reaction kinetics rather than achieving full conversion. This is because the conversion in industrial reactions depends not only on intrinsic reaction kinetics but also on factors such as reactor geometry, fluid dynamics, transport phenomena, and space velocity. Based on these indications, this work assumes full conversion of ammonia in the cracking unit, with only trace levels of ammonia present in the outlet stream, consistent with assumptions made in other energy studies.^17,47−49,57^

The reactor representing the FC is modeled as an R-STOIC reactor, where the reaction and the corresponding conversion are explicitly defined.^51^ This reactor simulates the reaction between hydrogen and oxygen to produce water and generate energy. The block is fed by two streams: the hydrogen/nitrogen stream (for the PEMFC case) or ammonia stream (for the SOFC case), and an auxiliary air stream. The air stream is preheated in a heat exchanger with water produced by the FC. Under the design conditions for configurations with HT-PEMFC, operating at 160 °C with a 75/25 H_2_/N_2_ ratio, nitrogen separation can be omitted without causing a significant drop in efficiency (Table 1). Consequently, the primary focus shifts to the removal of trace amounts of NH_3_ from the reactor outlet mixture, as these significantly impact FC performance. As discussed in the introduction, literature findings indicate that commercially available adsorbents can effectively reduce ammonia traces to levels compatible with FC operations.^20^ For configurations using LT-PEMFC, the design accounts for an efficiency loss of approximately 20% due to the presence of N_2_ (Table 2), as supported by findings discussed in the introduction section.^37^ Heat exchangers were modeled in Aspen with an exchange efficiency of 80%. For reference, the maximum achievable theoretical efficiency was evaluated for each configuration, assuming a *U*_f_ of 100%. Subsequently, calculations were repeated for a more realistic *U*_f_ of 80%. Under this scenario, additional heat could be recovered from unreacted residual hydrogen with the adoption of an afterburner to sustain endothermic units within the integrated process. In the context of fuel cells, unconverted hydrogen is rarely recycled because it would be too diluted for effective reuse in these devices. While separation technologies could indeed be integrated, hydrogen/nitrogen separation is primarily performed using PSA (Pressure Swing Adsorption) technology.^58^ This method requires gas compression and incurs energy losses associated with the regeneration of adsorbent beds, thereby reducing the overall efficiency of the system, especially when dealing with highly diluted hydrogen. For these reasons, this aspect was not included in the present study.

### 2.2 Configurations

Four main process configurations were analyzed using different types of FCs: HT-PEMFC, LT-PEMFCs, direct-ammonia SOFC, and hydrogen SOFC. Parametric simulations were performed across a range of FC efficiencies commonly observed in practice (50–65%). For each configuration, the initial ammonia flow rate supplied to the system was calculated to produce 1 kW of electrical power, considering the FC efficiency, assuming no heat losses, and a *U*_f_ of 100%, according to eq 1 (energetic and material balances are fully scalable).
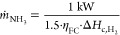
1where *ṁ*_NH_3__ is the ammonia molar flow rate, η_FC_ the FC efficiency, and Δ*H*_c,H_2__ is the lower heat value of hydrogen.

The parameters adopted for the simulations are summarized in Table S1 (Supporting Information).

#### 2.2.1 Configuration 1: HT-PEMFC-Based System

**Figure 2:**
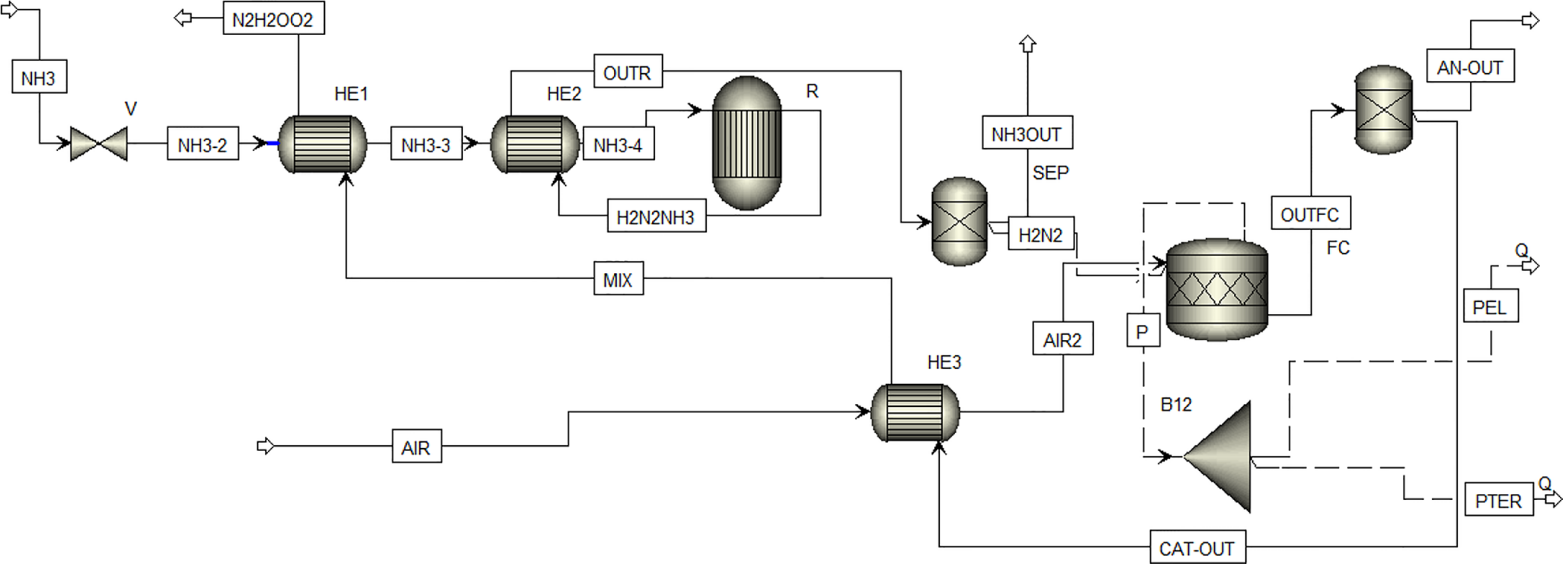
Configuration 1 (Aspen scheme), HT-PEMFC-based system.

The first configuration includes the following components (Figure 1a, Aspen Scheme Figure 2):

- Ammonia storage: ammonia is stored at 8.6 bar and 20 °C in its liquid state.
- Lamination: ammonia undergoes lamination to 1 bar, resulting in a temperature of −33 °C and a vapor fraction of 0.21
- First preheating (HE1): Energy recovery occurs during the first preheating stage, raising the ammonia to −33 °C with vapor fraction of 0.93
- Second preheating (HE2): Additional energy recovery takes place during the second preheating stage at 526–556 °C, depending on the *U*_f_.
- Cracking reactor: Ammonia is decomposed at 600 °C
- Downstream cooling (recovery): The mixture is cooled to at 160 °C for further energy recovery.

The heat required to preheat air from 20 to 160 °C is provided by additional energy recovery from the water and nitrogen mixture exiting the FC (HE3, Figure 2). For a *U*_f_ of 80%, additional heat can be recovered from unreacted residual hydrogen to sustain endothermic units within the system.

#### 2.2.2 Configuration 2: LT-PEMFC-Based System

This configuration (Figure 1a, Aspen Scheme Figure 2) adapts LT-PEMFCs at 80 °C, following a similar approach to the HT-PEMFC-based system. Both the reference case of a 100% *U*_f_ and a more realistic 80% *U*_f_ were analyzed. As highlighted in the introduction section, a 20% efficiency loss is considered due to the presence of 25% nitrogen in the feeding mixture.^36,37^ The operating conditions for this configuration are as follows:

- Ammonia storage: Ammonia is stored at 8.6 bar and 20 °C in its liquid state.
- Lamination: Ammonia undergoes lamination at 1 bar, reducing the temperature to −33 °C with vapor fraction of 0.21.
- First preheating: Energy recovery during the first preheating stage increases the vapor fraction to 0.62 while maintaining the temperature at −33 °C.
- Second preheating: Further energy recovery raises the temperature to 490–520 °C.
- Cracking reactor: Ammonia is decomposed in a cracking reactor at 600 °C.
- Downstream cooling (recovery): the mixture is cooled to at 80 °C, enabling additional energy recovery.

The heat required to preheat air from 20 to 80 °C is supplied by energy recovery from the water and nitrogen mixture exiting the FC.

#### 2.2.3 Configuration 3: Direct-Ammonia-SOFC-Based System

**Figure 3:**
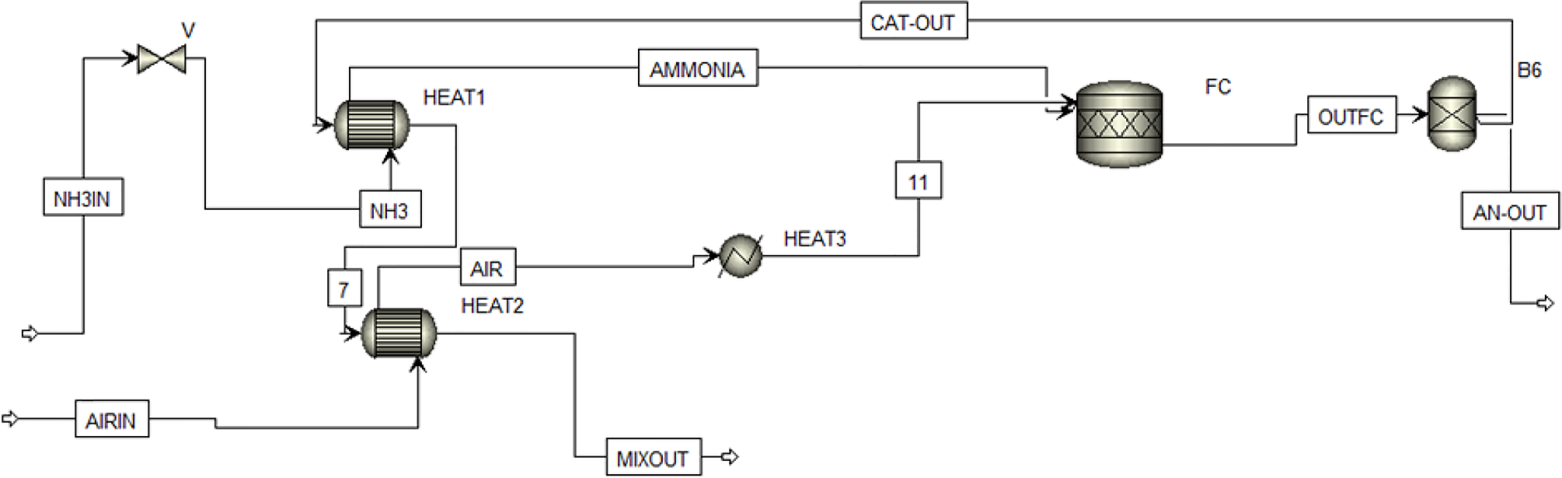
Configuration 3 (Aspen scheme), direct-ammonia-SOFC-based system. When *U*_f_ = 100% Heat exchanger 3 (HEAT3) is not present.

*Configuration 3* (Figure 1b, Aspen Scheme Figure 3) employs a direct ammonia SOFC as the core component. The steps and operating conditions are as follows:

- Ammonia storage: Ammonia is stored at 8.6 bar and 20 °C in its liquid state.
- Lamination: Ammonia undergoes lamination to 1 bar
- Preheating (HE1): Ammonia is preheated using energy recovery, raising its temperature to 700 °C.
- SOFC Operation: The SOFC operates at 700 °C.
- Downstream cooling (heat recovery): the exhaust stream is cooled from 700 to 400 °C, with the heat recovery integrated into the process.

In the case of a 100% *U*_f_, the heat required to preheat the incoming air from 20 to 383 °C is sourced through additional energy recovery as the mixture is cooled from 400 to 140 °C (HE2). However, since air can only be preheated up to 383 °C, an associated energy loss occurs within the FC due to incomplete thermal integration. In the case of an 80% *U*_f_, residual hydrogen can further preheat the air to 700 °C (HE3), with no associated energy loss.

#### 2.2.4 Configuration 4: Hydrogen-SOFC-Based System

**Figure 4:**
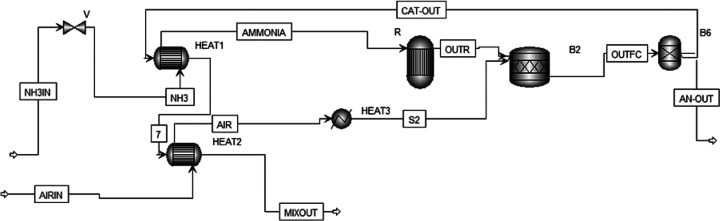
Configuration 4 (Aspen scheme), hydrogen-SOFC-based system. When *U*_f_ = 100% heat exchanger 3 (HEAT3) is not present.

*Configuration 4* (Figure 1c, Aspen Scheme Figure 4) utilizes a hydrogen-SOFC as the core component. The system operates through the following steps and conditions:

- Ammonia storage: Ammonia is stored at 8.6 bar and 20 °C in its liquid state.
- Lamination: Ammonia undergoes lamination to 1 bar.
- Preheating (HE1): Ammonia is preheated using energy recovery, raising its temperature to 600 °C.
- Cracking reactor: Ammonia is decomposed in a cracking reactor at 600 °C, with the process thermally sustained by the heat dissipated from the FC.^45^
- SOFC Operation: The SOFC operates at 700 °C.
- Downstream cooling (heat recovery): the exhaust stream is cooled from 700 to 430 °C, with the heat recovery integrated into the process.

For a 100% *U*_f_, the heat required to preheat the incoming air from 20 to 383 °C is sourced through additional energy recovery as the mixture is cooled from 400 to 148 °C (HE2). However, since air can only be preheated up to 383 °C, an associated energy loss occurs within the FC due to incomplete thermal integration. In the case of an 80% *U*_f_, residual hydrogen can further preheat the air to 700 °C (HE3), with no associated energy loss.

### 2.3 Key Performance Indicators

Based on the results of the simulations, key metrics such as system efficiency, autothermal behavior, and CO_2_ from external combustion (used to sustain endothermic units) were evaluated for each configuration. Overall system efficiency was defined as the ratio between the electrical output and the total energy fed into the system^50^ (i.e., the inherent energy in the fuel and any external thermal energy supplied):
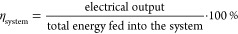
2

Considering the methane combustion reaction, the amount of CO_2_ produced per kWh of electricity generated by the system to sustain the cracking reactor was estimated. Knowing that CH_4_ combustion releases approximately 0.20 kg of CO_2_ per thermal kWh (kg_CO2_/kWh = 0.20) and considering a boiler efficiency of 0.90, the CO_2_ released is calculated as
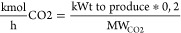
3
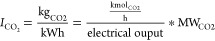
4

In addition, we calculated the autothermal efficiency to evaluate the system thermal self-sufficiency as follows:
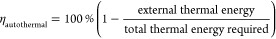
5where η_autothermal_ is the autothermal efficiency, and “external thermal energy” refers to the heat energy supplied from external sources.

## 3 Results

### 3.1 Configuration 1: HT-PEMFC-Based System

In Configuration 1, with total hydrogen utilization (*U*_f_ = 100%), the system is not autothermal as it recovers 0.31 kWh and requires an additional 0.32 kWh of thermal power from an external source, such as methane combustion. This setup results in the release of 1.60 × 10^–3^ kmol/h of CO_2_ per electrical kWh, equivalent to 0.07 kg CO_2_/kWh. With a FC efficiency of 50.0%, this configuration achieves autothermal and overall system efficiencies of 48 and 43.1%, respectively.

The preheating of the reactants is thermally sustained by internal sources, but the ammonia decomposition reactor still needs further energy. Specifically, partial vaporization of ammonia from vapor fraction = 0.21 to vapor fraction = 0.93 (at −33 °C) requires 0.10 kWh, which is provided by cooling the H_2_O/N_2_/O_2_ mixture from HE3 (Figure 1a) from 75 to 65 °C. Heating ammonia to 556 °C requires 0.14 kWh, which is supplied by cooling the H_2_/N_2_/NH_3_ mixture exiting the reactor from 600 to 160 °C (FC inlet temperature). Heating air from 20 to 160 °C requires 0.06 kWh, achieved by cooling the H_2_O/N_2_/O_2_ mixture leaving the FC from 160 to 75 °C. The simulation software accounts for water condensation at temperatures below 100 °C.

Despite these internal heat recovery strategies, the ammonia decomposition reactor still requires an additional 0.32 kWh of thermal power from an external source.

When considering a *U*_f_ of 80%, partial hydrogen consumption introduces a slight penalty reduction in both efficiency and electrical power. However, using 20% of unreacted hydrogen from the anode outlet stream to thermally sustain the cracking reactor eliminates the need for additional external thermal power, achieving autothermal operation (η_autothermal_ = 100%) and reducing associated CO_2_ emissions. This configuration delivers an electrical output of 0.80 kWh with an overall system efficiency of 40.2%, based on an average FC efficiency of 50.0%. This is slightly lower than the 1 kWh and 43.1% efficiency achieved under maximum hydrogen utilization.

The autothermal nature of the system at the more realistic *U*_f_ provides significant advantages in energy self-sufficiency and carbon footprint reduction. By optimizing internal heat recovery and hydrogen utilization strategies, this configuration offers a more sustainable and efficient alternative, despite the minor trade-offs in power output and efficiency.

### 3.2 Configuration 2: LT-PEMFC-Based System

Similar to Configuration 1, when hydrogen is entirely consumed, this system is not autothermal. Considering a FC efficiency of 50.0%, the autothermal and a overall system efficiencies are 42.0 and 34.3%, respectively. Specifically, partial vaporization of ammonia from vapor fraction = 0.21 to vapor fraction = 0.62 (at −33 °C) requires 0.05 kWh, which is supplied by cooling the gas mixture exiting HE3 (Figure 1) from 75 to 70 °C. HE3 also functions as the FC inlet air preheater. Raising the ammonia temperature to 490 °C requires 0.17 kWh, supplied by cooling the gas mixture exiting the reactor from 600 to 80 °C (FC inlet temperature).

Despite these internal heat recovery measures, the ammonia decomposition reactor still requires an additional 0.33 kWh of thermal power. This is supplied by burning additional methane, resulting in the release of 1.70 × 10^–3^ kmol/h of CO_2_ per electrical kWh, equivalent to 0.07 kg CO_2_/kWh.).

In contrast, a lower *U*_f_ (80%) eliminates the need for external heat power (η_autothermal_ = 100%), reducing emissions. This configuration delivers an electrical output of 0.64 kWh and an overall system efficiency of 31.2%, compared to the 0.80 kWh output expected with the total H_2_ consumption under the same configuration (overall system efficiency of 34.30%). The reduced efficiency is mainly due to the impact of nitrogen dilution on LT-PEMFC performance.

### 3.3 Configuration 3: Direct-Ammonia-SOFC-Based System

In the case of the direct-ammonia SOFC, the typical FC efficiency is comparable to, or slightly higher than, hydrogen-fueled cells. For this reason, a typical value of 55% was adopted.^59−61^ This configuration is autothermal for both *U*_f_ values (η_autothermal_ = 100%) due to the high operating temperature, which enables the recovery of high-quality heat. In the case of complete H_2_ consumption, the heat required to preheat ammonia to 700 °C (0.24 kWh) is recovered by cooling the gas mixture exiting the FC from 700 to 400 °C. Additionally, the heat needed to raise air temperature from 20 to 383 °C is derived from further energy recovery (0.20 kWh) by cooling the mixture from 400 to 140 °C. Under these conditions, the system is autothermal and achieves a power output of 0.78 kWh, with an overall efficiency of 43%.

In the case of partial hydrogen consumption (*U*_f_ = 80%), the overall system efficiency is reduced to 39%. Although the FC produces less electrical energy, unconverted hydrogen is combusted to preheat the air to 700 °C, enabling additional energy recovery and maintaining the system autothermal behavior.

### 3.4 Configuration 4: Hydrogen-SOFC-Based System

For the hydrogen-powered SOFC, the typical FC efficiency is 55%. This configuration is autothermal for both values of the *U*_f_ (η_autothermal_ = 100%). The heat required to preheat ammonia to 600 °C (0.21 kWh) is recovered by cooling the gas mixture exiting the FC from 700 to 430 °C. Additionally, the heat needed to raise air temperature from 20 to 383 °C is sourced through further energy recovery (0.20 kWh) by cooling the mixture from 430 to 148 °C. The heat to support the ammonia cracking (0.31 kWh) is supplied by the SOFC dissipated heat. The electrical energy produced (0.77–0.70) depends on the *U*_f_ and is slightly lower than in the direct-ammonia SOFC case because some energy is used to heat the reactor outlet mixture from 600 to 700 °C. For *U*_f_ = 100%, the system is autothermal and achieves a power output of 0.77 kWh, with an overall efficiency of 42.5%. For partial hydrogen consumption (*U*_f_ = 80%), the overall system efficiency decreases to 38.5%. Although the SOFC produces less electrical energy, unconverted hydrogen is combusted to preheat the air to 700 °C, allowing for additional energy recovery and maintaining the system autothermal behavior.

### 3.5 Summary and Comparison

**Figure 5:**
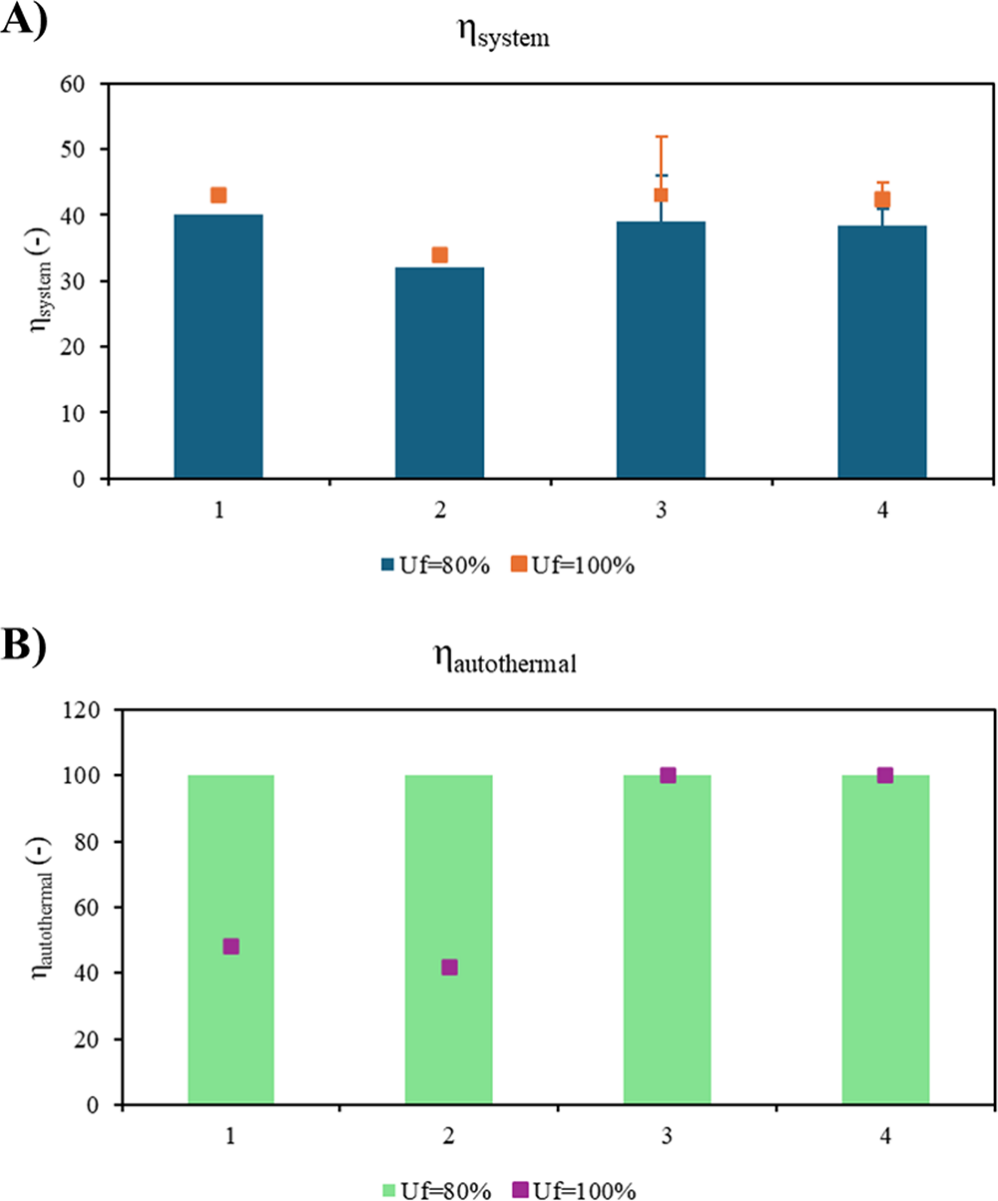
(A) Overall efficiency η_system_, (B) autothermal efficiency η_autothermal_ of different process configurations. Error bars reflect the wider fuel cell efficiency reported in the literature for SOFC.

Figure 5 shows the performance of the different configurations, considering average efficiency values for the specific FC types and comparing two operating conditions: *U*_f_ = 80% and theoretical reference *U*_f_ = 100%. In general, the overall efficiency of the system increases with the *U*_f_ (Figure 5A). Configurations 1, 3, and 4 exhibit comparable overall efficiency, while Configuration 2 is characterized by the lowest performance. For Configurations 3 and 4, error bars representing the overall efficiency are included for both *U*_f_ values, accounting for the broader range of FC efficiencies reported in the literature, i.e., 0.55–0.65 and 0.55–0.60 for Configuration 3 and 4, respectively.^59−61^ Notably, higher FC efficiency translate into improved overall system efficiencies. Specifically, for Configuration 3, adopting a more efficient direct-ammonia SOFC enhances the overall system efficiency by approximately 7% under partial hydrogen utilization and 9% under complete hydrogen consumption.

Notably, all configurations achieve autothermal operation under partial consumption, with no CO_2_ emissions from external fuel combustion, as the unconverted hydrogen is utilized to thermally support the ammonia decomposition reactor (Figure 5B). Conversely, under complete hydrogen consumption, integrated systems with HT-PEMFCs and LT-PEMFCs are not autothermal, requiring more than 50% of the total system heat to be supplied by external sources, which results in CO_2_ emissions. It is important to emphasize that autothermal conditions can also be achieved using ammonia-fueled crackers. These systems are generally advantageous in terms of energy efficiency, exergy performance, and economic feasibility when evaluated as standalone units, as they reduce reactor volumes and heat requirements.^62^ However, in the context of integrated systems with fuel cells (FCs), their adoption may decrease overall energy efficiency, particularly at lower utilization factor values.As a case study and proof of concept, the results of calculations conducted for integration with HT-PEMFCs are presented in Table S4 (Supporting Information).

Considering the typical variability in FC efficiencies,^55^ the analysis was extended to encompass different conditions. Table 3 summarizes the parametric analysis conducted at varying FC efficiencies for the four proposed configurations (Figures 2–4). Details of streamflow rates and compositions are provided in Tables S2 and S3 (Supporting Information). To summarize, under a *U*_f_ of 80% and for a given FC efficiency (e.g., 0.50), HT-PEMFC-based configurations achieve the highest overall efficiency (40.2%), followed by configurations with direct-ammonia SOFCs (39%) and hydrogen SOFCs (38.5%). LT-PEMFC-based configurations exhibit the lowest overall efficiency (31.2%), primarily due to the negative impact of nitrogen dilution. However, as previously highlighted, different FC types generally exhibit varying average efficiencies, which allows SOFCs to remain competitive, under the given conditions. The analysis underscores the importance of advancing FC materials to enhance performance and increase the overall system efficiency of integrated systems. Moreover, autothermal operation is achievable across all configurations at average levels of hydrogen consumption. Future research address the effects of FCs degradation and lifespan to provide a more comprehensive evaluation and comparison. In light of the current analysis and the fragmented insights from the literature discussed in the introduction, HT-PEMFCs and hydrogen SOFCs may offer an optimal balance between degradation rates, durability, nitrogen dilution tolerance, and overall efficiency. Table 4 provides a summary of the key characteristics of the analyzed system configurations.

**Table 3:** Parametric Analysis at Different FC Efficiencies

η_FC_ (%)	molar flow of ammonia ()	electrical output (kWh)	heat flow required (kWh)	η_system_ (%)	η_autothermal_ (%)	*I*_CO_2__ ()
*Configuration 1 (U*_*f*_*= 100%)*						
50.0	2.0 × 10^–2^	1.00	0.32	43.1	48	0.07
55.0	1.8 × 10^–2^		0.29	47.4		0.06
60.0	1.7 × 10^–2^		0.27	51.5		0.05
*Configuration 1 (U*_*f*_*= 80%)*						
50.0	2.0 × 10^–2^	0.80	0	40.2	100	0
55.0	1.8 × 10^–2^			44.3		
60.0	1.7 × 10^–2^			48.3		
*Configuration 2 (U*_*f*_*= 100%)*						
50.0	2.0 × 10^–2^	0.8	0.33	34.3	42	0.07
55.0	1.8 × 10^–2^		0.31	37.7		0.07
60.0	1.7 × 10^–2^		0.28	41		0.06
*Configuration 2 (U*_*f*_*= 80%)*						
50.0	2.0 × 10^–2^	0.64	0	31.2	100	0
55.0	1.8 × 10^–2^		0	34.9		
60.0	1.7 × 10^–2^		0	38.1		
*Configuration 3 (U*_*f*_*= 100%)*						
50.0	2.0 × 10^–2^	0.78	0	39.0	100	0
55.0	1.8 × 10^–2^			43.0		
60.0	1.7 × 10^–2^			46.0		
65.0	1.5 × 10^–2^			52.0		
*Configuration 3 (U*_*f*_*= 80%)*						
50.0	2.0 × 10^–2^	0.71	0.20	36.0	100	0
55.0	1.8 × 10^–2^		0.18	39.0		
60.0	1.7 × 10^–2^		0.17	42.0		
65.0	1.5 × 10^–2^		0.16	46.0		
*Configuration 4 (U*_*f*_*= 100%)*						
50.0	2.0 × 10^–2^	0.77	0	38.5	100	0
55.0	1.8 × 10^–2^			42.5		
60.0	1.7 × 10^–2^			45.0		
*Configuration 4 (U*_*f*_*= 80%)*						
50.0	2.0 × 10^–2^	0.70	0.20	35.0	100	0
55.0	1.8 × 10^–2^		0.18	38.5		
60.0	1.7 × 10^–2^		0.17	41.0		

**Table 4:** Summary Table of Investigated Configurations

configuration	*U*_f_	fuel cell	advantages	disadvantages
1	theoretical 100%	HT-PEMFC	maximum *U*_f_ results in the highest achievable total system efficiency (51.5%)	nonautothermal behavior; a shorter lifespan compared to LT-PEMFCs
			better suited for portable applications	
	effective 80%	HT-PEMFC	autothermal behavior; 20% of the unreacted hydrogen from the fuel cell supports cracking	overall efficiency reduced by 3.2 percentage points compared to the maximum obtainable value. A shorter lifespan compared to LT-PEMFCs
			better suited for portable applications	
2	theoretical 100%	LT-PEMFC	maximum *U*_f_ results in the highest achievable total system efficiency (41%)	nonautothermal behavior; nitrogen dilution in LT-PEMFCs significantly decreases efficiency
			better suited for portable applications	
	effective 80%	LT-PEMFC	autothermal behavior; 20% of the unreacted hydrogen from the fuel cell supports cracking	overall efficiency reduced by 2.9 percentage points compared to the maximum obtainable value. Nitrogen dilution in LT-PEMFCs significantly reduces efficiency
			better suited for portable applications	
3	theoretical 100%	direct-ammonia SOFC	maximum *U*_f_ results in the highest achievable total system efficiency (46.0–52.0%); Autothermal behavior	produces less electrical energy as air enters at a lower temperature than operating temperature; higher degradation rates and a lower TRL
			better suited for stationary applications	
	effective 80%	direct-ammonia SOFC	autothermal behavior	overall efficiency reduced by 4 to 6 percentage points compared to the maximum obtainable value
			better suited for stationary applications	higher degradation rates and a lower TRL
4	theoretical 100%	hydrogen SOFC	maximum *U*_f_ results in the highest achievable total system efficiency (42.5–46.0%); Autothermal behavior	produces less electrical energy as reactants enter at a lower temperature than operating temperature
			better suited for stationary applications	higher TRL than direct-ammonia SOFC
	effective 80%	hydrogen SOFC	autothermal behavior	overall efficiency reduced by 4 percentage points compared to the maximum obtainable value
			better suited for stationary applications	higher TRL than direct-ammonia SOFC

## 4 Conclusions

The latest projections from the International Renewable Energy Agency highlight that the adoption of hydrogen technologies and improved energetic efficiency in integrated systems will be key factors in transitioning to a sustainable energy economy. Leveraging the existing large-scale ammonia infrastructure, the use of ammonia as a hydrogen carrier in integrated systems with FC offers a practical short-term solution that can be implemented promptly. Various process configurations were analyzed from an energy perspective to assess heat recovery, overall system efficiency, and emissions associated with thermically sustaining different ammonia-based systems. These analyses considered FC efficiencies ranging from 50.0 to 65.0%, depending on the system. The results indicate that the overall energy performance of the system is primarily dependent on FC efficiency and the extent of heat recovery. Notably, utilizing the enthalpic content of up to 20% unreacted hydrogen to achieve full autothermal operation of the integrated system is an attractive approach. While this slightly decreases overall efficiency, it significantly reduces emissions and simplifies system design. The adoption of LT-PEMFCs is notably impacted by nitrogen dilution of hydrogen fuel, with overall efficiencies reaching only up to 34.3% across various configurations when assuming an average FC efficiency of 50.0%. A direct comparison between HT-PEMFCs-based and SOFC-based systems reveals higher overall energy efficiencies, approximately 42.0%, when considering an average FC efficiency of 50.0% for HT-PEMFC and 55.0% for direct-ammonia and hydrogen SOFC. Published results on technological readiness and components lifespans suggest that HT-PEMFCs and hydrogen SOFC may represent a promising technology for such integrated systems. Although they still face limitations, these technologies demonstrate lower degradation rates and longer lifespans compared to direct-ammonia SOFC. HT-PEM offer the advantage of less extreme operating conditions, making them more suitable for mobile applications, whereas SOFC better suited for stationary applications. Future research will focus on the energy implications of separation technologies, their impact on the overall balance of plant, and detailed temperature profiles of cracking reactors to evaluate the influence of power scale on system efficiency.

## Supporting Information Available

Conditions adopted for process simulations and stream table (PDF)

## List of Symbols

MW_CO2_: molecular weight of CO_2_
I_CO2_: mass of CO_2_ per kWh of produced electrical energy
η_autothermal_: autothermal efficiency
η_system_: overall system efficiency
η_FC_: fuel cell efficiency
*ṁ*_NH_3__: molar flow rate of ammonia entering the system
Δ*H*_c,H_2__: calorific heat value of hydrogen
*U*_f_: utilization factor

## List of Abbreviations

FC: fuel cell
PEMFC: proton exchange membrane fuel cell
LT-PEMFC: low-temperature proton exchange membrane fuel cell
HT-PEMFC: high-temperature proton exchange membrane fuel cell
SOFC: solid oxide fuel cell
