# Supplementary material for: Ammonia as a Hydrogen Carrier: Energetic Assessment of Processes Integrated with Fuel Cells for Power Generation
Source: Energy Fuels. 2025 Jan 24;39(5):2843–53. doi: 10.1021/acs.energyfuels.4c04626 (PMC11809026; doi:10.1021/acs.energyfuels.4c04626)
Supplement: Supplementary file 1 — ef4c04626_si_001.pdf [file ef4c04626_si_001.pdf]

Ammonia as a hydrogen carrier: energetic assessment of processes integrated with fuel cells for power generation

Maria Portarapillo†, Augusto Bellucci Sessa†, Danilo Russo*, Almerinda di Benedetto

Department of Chemical Engineering, Materials, and Industrial Production, University of Naples Federico II, P.le V. Tecchio 80, 80125, Napoli, Italy.

† equal contribution

* corresponding author: Danilo Russo, danilo.russo3@unina.it

Table S1. Conditions adopted for process simulations

η_{FC}	**Molar flow of ammonia ($\frac{kmol_{NH3}}{h}$)**	**T_{IN} R (C°)**	**T_{IN} F_C (C°)**	**$T_{IN\text{-}air}$ F_C (C°)**	**HE1 (kWh)**	**HE2 (kWh)**	**HE3 (kWh)**	**P (bar)**
Configuration 1 ($U_f = 100\%$)								
0.50	$2.0*10^{-2}$	556	160	160	0.10	0.14	0.06	1
0.55	$1.8*10^{-2}$	566	160	160	0.09	0.13	0.06	1
0.60	$1.7*10^{-2}$	576	160	160	0.08	0.12	0.06	1
Configuration 1 ($U_f = 80\%$)								
0.50	$2.0*10^{-2}$	530	160	160	0.09	0.14	0.06	1
0.55	$1.8*10^{-2}$	540	160	160	0.08	0.13	0.06	1
0.60	$1.7*10^{-2}$	550	160	160	0.07	0.12	0.06	1
Configuration 2 ($U_f = 100\%$)								
0.50	$2.0*10^{-2}$	490	80	80	0.05	0.17	0.01	1
0.55	$1.8*10^{-2}$	500	80	80	0.04	0.16	0.01	1
0.60	$1.7*10^{-2}$	510	80	80	0.04	0.15	0.01	1
Configuration 2 ($U_f = 80\%$)								
0.50	$2.0*10^{-2}$	460	80	80	0.04	0.17	0.01	1
0.55	$1.8*10^{-2}$	470	80	80	0.03	0.16	0.01	1
0.60	$1.7*10^{-2}$	480	80	80	0.03	0.15	0.01	1
Configuration 3 ($U_f = 100\%$)								
0.50	$2.0*10^{-2}$	-	700	383	0.28	0.22	-	1
0.55	$1.8*10^{-2}$		700	383	0.24	0.20		1
0.60	$1.7*10^{-2}$		700	383	0.20	0.18		1
0.65	$1.5*10^{-2}$		700	383	0.16	0.16		1
Configuration 3 ($U_f = 80\%$)								
0.50	$2.0*10^{-2}$	-	700	700	0.28	0.22	0.20	1
0.55	$1.8*10^{-2}$		700	700	0.24	0.20	0.18	1
0.60	$1.7*10^{-2}$		700	700	0.20	0.18	0.16	1
0.65	$1.5*10^{-2}$		700	700	0.16	0.16	0.14	1
Configuration 4 ($U_f = 100\%$)								
0.50	$2.0*10^{-2}$	600	600	383	0.28	0.22		1

0.55	$1.8*10^{-2}$	600	600	383	0.24	0.20	-	1
0.60	$1.7*10^{-2}$	600	600	383	0.20	0.18		1
Configuration 4 (U_f = 80%)								
0.50	$2.0*10^{-2}$	600	600	700	0.28	0.22	0.20	1
0.55	$1.8*10^{-2}$	600	600	700	0.24	0.20	0.18	1
0.60	$1.7*10^{-2}$	600	600	700	0.20	0.18	0.16	1

Table S2. Composition of the main streams evaluated. U_f = utilization factor

Composition (Molar fraction)	NH3in	Airin	OUTR	INFC	AN-OUT	CAT-OUT
Configuration 1 ($U_f = 100\%$)	NH3	AIR	$0.7496H_2$ $0.2498N_2$ $0.0006NH_3$	$0.75H_2$ $0.25N_2$	N_2	$0.001O_2$ $0.65N_2$ $0.349H_2O$
Configuration 1 ($U_f = 80\%$)	NH3	AIR	$0.7496H_2$ $0.2498N_2$ $0.0006NH_3$	$0.75H_2$ $0.25N_2$	$0.375H_2$ $0.625N_2$	$0.034O_2$ $0.676N_2$ $0.29H_2O$
Configuration 2 ($U_f = 100\%$)	NH3	AIR	$0.7496H_2$ $0.2498N_2$ $0.0006NH_3$	$0.75H_2$ $0.25N_2$	N_2	$0.033O_2$ $0.65N_2$ $0.26H_2O$
Configuration 2 ($U_f = 80\%$)	NH3	AIR	$0.7496H_2$ $0.2498N_2$ $0.0006NH_3$	$0.75H_2$ $0.25N_2$	$0.375H_2$ $0.625N_2$	$0.034O_2$ $0.676N_2$ $0.29H_2O$
Configuration 3 ($U_f = 100\%$)	NH3	AIR	-	NH3	N_2	$0.003O_2$ $0.69N_2$ $0.307H_2O$
Configuration 3 ($U_f = 80\%$)	NH3	AIR	-	NH3	$0.375H_2$ $0.625N_2$	$0.003O_2$ $0.707N_2$ $0.29H_2O$
Configuration 4 ($U_f = 100\%$)	NH3	AIR	$0.7496H_2$ $0.2498N_2$ $0.0006NH_3$	$0.7496H_2$ $0.2498N_2$ $0.0006NH_3$	N2	$0.003O_2$ $0.69N_2$ $0.307H_2O$
Configuration 4 ($U_f = 80\%$)	NH3	AIR	$0.7496H_2$ $0.2498N_2$ $0.0006NH_3$	$0.7496H_2$ $0.2498N_2$ $0.0006NH_3$	$0.375H_2$ $0.625N_2$	$0.003O_2$ $0.69N_2$ $0.307H_2O$

Table S3. Molar flow rate of the main streams evaluated. U_f = utilization factor; η_{FC} = overall system efficiency.

Molar flow (kmol/h)<br>$\eta_{FC} = 0.5$<br>$\eta_{FC} = 0.55$<br>$\eta_{FC} = 0.6$	NH3in	Airin	OUTR	INFC	AN-OUT	CAT-OUT
Configuration 1 ($U_f = 100\%$)	0.02<br>0.018<br>0.017	0.072<br>0.065<br>0.06	0.04<br>0.036<br>0.034	0.04<br>0.036<br>0.034	0.01<br>0.009<br>0.0085	0.087<br>0.078<br>0.074
Configuration 1 ($U_f = 80\%$)	0.02<br>0.018<br>0.017	0.072<br>0.065<br>0.06	0.04<br>0.036<br>0.034	0.04<br>0.036<br>0.034	0.016<br>0.0145<br>0.0135	0.08<br>0.072<br>0.68
Configuration 2 ($U_f = 100\%$)	0.02<br>0.018<br>0.017	0.072<br>0.065<br>0.06	0.04<br>0.036<br>0.034	0.04<br>0.036<br>0.034	0.01<br>0.009<br>0.0085	0.087<br>0.078<br>0.074
Configuration 2 ($U_f = 80\%$)	0.02<br>0.018<br>0.017	0.072<br>0.065<br>0.06	0.04<br>0.036<br>0.034	0.04<br>0.036<br>0.034	0.016<br>0.0145<br>0.0135	0.08<br>0.072<br>0.68
Configuration 3 ($U_f = 100\%$)	0.02<br>0.018<br>0.017	0.072<br>0.065<br>0.06	-	0.02<br>0.018<br>0.017	0.01<br>0.009<br>0.0085	0.087<br>0.078<br>0.074
Configuration 3 ($U_f = 80\%$)	0.02<br>0.018<br>0.017	0.072<br>0.065<br>0.06	-	0.02<br>0.018<br>0.017	0.016<br>0.0145<br>0.0135	0.08<br>0.072<br>0.68
Configuration 4 ($U_f = 100\%$)	0.02<br>0.018<br>0.017	0.072<br>0.065<br>0.06	0.04<br>0.036<br>0.034	0.04<br>0.036<br>0.034	0.01<br>0.009<br>0.0085	0.087<br>0.078<br>0.074
Configuration 4 ($U_f = 80\%$)	0.02<br>0.018<br>0.017	0.072<br>0.065<br>0.06	0.04<br>0.036<br>0.034	0.04<br>0.036<br>0.034	0.016<br>0.0145<br>0.0135	0.08<br>0.072<br>0.68

Table S4. Calculated efficiency for ammonia-fueled-cracker-based integrated systems.

**η_{FC} (%)**	**Molar flow of ammonia ($\frac{kmol_{NH3}}{h}$)**	**Electrical output (kW)**	**Heat flow required (kW)**	**η_{system} (%)**
Configuration 1. Ammonia fueled. Utilization factor = 100%				
50.0	$2.0*10^{-2}$	0.85	0.29	37.0
55.0	$1.8*10^{-2}$		0.26	41.0
60.0	$1.7*10^{-2}$		0.24	44.0
Configuration 1. Ammonia fueled. Utilization factor = 80%				
50.0	$2.0*10^{-2}$	0.68	0	34.0
55.0	$1.8*10^{-2}$			37.0
60.0	$1.7*10^{-2}$			41.0
